# Mitochondria-Derived Reactive Intermediate Species Mediate Asbestos-Induced Genotoxicity and Oxidative Stress–Responsive Signaling Pathways

**DOI:** 10.1289/ehp.1104287

**Published:** 2012-03-07

**Authors:** Sarah X.L. Huang, Michael A. Partridge, Shanaz A. Ghandhi, Mercy M. Davidson, Sally A. Amundson, Tom K. Hei

**Affiliations:** 1 Department of Environmental Health Sciences, Mailman School of Public Health,; 2 Center for Radiological Research, and; 3 Department of Radiation Oncology, College of Physicians and Surgeons, Columbia University, New York, New York, USA

**Keywords:** asbestos, DNA oxidative damage, genotoxicity, mitochondria, oxidative stress–responsive signaling pathways, reactive oxygen species

## Abstract

Background: The incidence of asbestos-induced human cancers is increasing worldwide, and considerable evidence suggests that reactive oxygen species (ROS) are important mediators of these diseases. Our previous studies suggested that mitochondria might be involved in the initiation of oxidative stress in asbestos-exposed mammalian cells.

Objective: We investigated whether mitochondria are a potential cytoplasmic target of asbestos using a mitochondrial DNA–depleted (ρ^0^) human small airway epithelial (SAE) cell model: ρ^0^ SAE cells lack the capacity to produce mitochondrial ROS.

Methods: We examined nuclear DNA damage, micronuclei (MN), intracellular ROS production, and the expression of inflammation-related nuclear genes in both parental and ρ^0^ SAE cells in response to asbestos treatment.

Results: Asbestos induced a dose-dependent increase in nuclear DNA oxidative damage and MN in SAE cells. Furthermore, there was a significant increase in intracellular oxidant production and activation of genes involved in nuclear factor κB and proinflammatory signaling pathways in SAE cells. In contrast, the effects of asbestos were minimal in ρ^0^ SAE cells.

Conclusions: Mitochondria are a major cytoplasmic target of asbestos. Asbestos may initiate mitochondria-associated ROS, which mediate asbestos-induced nuclear mutagenic events and inflammatory signaling pathways in exposed cells. These data provide new insights into the molecular mechanisms of asbestos-induced genotoxicity.

Asbestos fibers are well-defined environmental and occupational carcinogens in humans and remain widely used in many developing countries (Nishikawa et al. 2008; Straif et al. 2009). Currently, the worldwide incidence of asbestos-induced cancer and other diseases is still on the rise because of their long latency periods (Nishikawa et al. 2008). Consequently, there is great urgency and importance in studying the molecular mechanisms underlying asbestos-related diseases for a better understanding of disease prevention and treatment strategy.

There is considerable evidence that asbestos-initiated chronic oxidative stress contributes to carcinogenesis and fibrosis by promoting oxidative DNA damage and regulating redox signaling pathways in exposed cells (Huang et al. 2011; Kamp and Weitzman 1999; Shukla et al. 2003; Toyokuni 2009; Xu et al. 1999, 2002). Asbestos fibers may initiate reactive oxygen species (ROS) production by multiple mechanisms. The surface iron associated with asbestos generates hydroxyl radicals either through a redox reaction or by catalyzing a Fenton-like reaction in exposed cells (Pezerat et al. 1989; Weitzman and Graceffa 1984). In addition, the uptake of asbestos fibers can stimulate phagocytic cells to release extracellular ROS through membrane-associated NADPH (nicotinamide adenine dinucleotide phosphate) oxidase (Kamp et al. 1992).

A possible important source of intracellular ROS generation in asbestos-exposed cells is mitochondrial oxidative phosphorylation. Mitochondria consume about 90% of the oxygen used by cells. In normal physiological conditions, mitochondrial oxidative phosphorylation represents the major site of endogenous oxidant generation. In pathophysiological conditions, certain chemical xenobiotics are known to use the mitochondrial respiratory chain to amplify reactive oxidant production (Gutierrez 2000). Two studies found that mitochondria-derived oxidative stress was a mediator of asbestos-induced apoptosis and cell toxicity in alveolar epithelial cells (Panduri et al. 2003, 2004).

Thus far, it is not clear whether mitochondrial-originated reactive oxidants mediate asbestos-induced nuclear DNA mutagenic events. Our previous study demonstrated that cytoplasmic components could initiate asbestos-induced oxidative stress and promote nuclear mutagenesis in A_L_ (human–hamster hybrid) cells (Xu et al. 2007). In the present study, we investigated whether mitochondria might be the potential cytoplasmic target of asbestos fibers. We examined the effects of chrysotile and crocidolite asbestos treatment on intracellular ROS production in human small airway epithelial (SAE) cells and found that mitochondrial-originated reactive oxidants were involved in mediating asbestos-induced nuclear DNA damage and mutagenesis. We also found that these oxidants altered the expression of 178 inflammation- and immune-related nuclear genes involved in signaling pathways known to be activated in response to oxidative stress in mammalian cells (Martindale and Holbrook 2002).

## Materials and Methods

*Asbestos fibers, cell culture, and treatments.* We used UICC (Union Internationale Contre le Cancer) standard reference samples of chrysotile and crocidolite asbestos in the present study. Stock solutions (1 mg/mL) were prepared as described previously (Xu et al. 2002). The human telomerase reverse transcriptase–immortalized human SAE cells were previously generated (Piao et al. 2005). Cells were maintained in complete small airway growth medium supplemented with growth factors (Lonza Group Ltd., Basel, Switzerland) at 37°C in a humidified 5% CO_2_ atmosphere. Typically, 70–80% confluent cell cultures were treated with chrysotile or crocidolite at concentrations of 0.5, 1, 2, and/or 4 µg/cm^2^ for 12, 24, or 48 hr. Hydrogen peroxide (H_2_O_2_) was added at final concentrations ranging from 100 to 500 µM for 30 min. In some experiments, 0.5% (vol/vol) dimethyl sulfoxide (DMSO; Sigma, St. Louis, MO) was added before and concurrently with asbestos treatment. Methods for assessing the toxicity of asbestos are described in Supplemental Material (http://dx.doi.org/10.1289/ehp.1104287).

*Mitochondrial-DNA (mtDNA)–depleted SAE cell generation.* The mtDNA–depleted (ρ^0^) SAE cell line was generated from the parental human SAE cells by ethidium bromide (EtBr) treatment, which is a standard method for generating ρ^0^ SAE cell lines from human cells (King and Attardi 1989). Cells were treated with 50 ng/mL EtBr for 2 months in ρ^0^ medium: complete small airway growth medium containing uridine (50 µg/mL), sodium pyruvate (1 mM), HEPES (20 mM), and glucose (4.5 g/L; all chemicals from Sigma). After this treatment, cells were maintained in ρ^0^ medium without EtBr. The ρ^0^ SAE cells generate energy through glycolysis using uridine and pyruvate supplements in the ρ^0^ media (King and Attardi 1989). The ρ^0^ status was verified by measuring mtDNA content and four mitochondrial functional markers: mitochondrial membrane potential, oxygen consumption rate, cytochrome c oxidase (COX) activity, and intracellular superoxide content [see Supplemental Material, pp. 3–4 (http://dx.doi.org/10.1289/ehp.1104287)].

*Immunofluorescence for 8-hydroxy-deoxyguanosine (8-OHdG) detection.* We determined oxidative DNA damage by measuring 8-OHdG levels using monoclonal antibody 1F7 (a gift from R. Santella, Columbia University). After 48 hr of treatment, control and treated cells were fixed and permeabilized following the protocol described previously (Partridge et al. 2007). Cells were incubated with 1F7 at 1:20 dilution for 1.5 hr at 37°C, followed by incubation with Alexa Fluor 488 goat anti-mouse IgG [Invitrogen (Life Technologies, Grand Island, NY)] for 30 min and propidium iodide (PI; BD Biosciences Pharmingen, San Diego, CA) for 10 min. Samples were visualized and images were captured on a confocal microscope (Nikon Eclipse TE2000-U; Nikon Corporation, Tokyo, Japan). The mean ± SD green fluorescence intensity per cell was obtained from approximately 200 cells per sample using Image-Pro Plus, version 6.0 (Media Cybernetics Inc., Bethesda, MD).

*Micronuclei (MN) induction.* Chromosomal damage of nuclear DNA was examined by assaying the frequency of MN (Fenech 2000). Control and asbestos-treated cells were incubated with 0.5 ng/µL cytochalasin B for 48 hr and then fixed and permeabilized. Cellular nuclei were stained with PI and cytoplasm was counterstained with Alexa Fluor 488 phalloidin (Invitrogen). Samples were visualized and images were captured with a fluorescence microscope [Olympus Bh-2 equipped with Olympus MicroSuite FIVE software (Olympus America, Center Valley, PA)]. About 500 binucleated cells per sample were examined, and cells with MN were scored manually. The percentage of cells containing MN was calculated for each sample.

*5´,6´-Chloromethyl-2´,7´dichlorodihydrofluorescein diacetate (CM-H_2_DCFDA).* The intracellular oxidant level was determined by CM-H_2_DCFDA (Invitrogen), a nonfluorescent probe that can be converted by oxidants into a green fluorescent product, CM-DCF (5´,6´-chloromethyl-2´,7´dichlorofluorescein). Parental and ρ^0^ SAE cells were treated with 0.5 µg/cm^2^ chrysotile for 24 hr. H_2_O_2_-exposed cells served as positive controls, and DMSO was used as a potential radical scavenger. Control and treated cells were labeled with Cell Tracker Red (Invitrogen) followed by 15 µM CM-H_2_DCFDA treatment for 45 min. Cells were immediately visualized and images were captured on the Nikon confocal microscope. All images were focused during brief illumination with a laser beam and collected with a single scan using a low laser power to avoid photooxidation of CM-H_2_DCFDA, which has been reported previously (Chen et al. 2005). The green fluorescence intensity was quantified by Image-Pro Plus and normalized to the Cell Tracker staining to account for the difference in cell thickness between the parental and ρ^0^ SAE cells. The mean ± SD fluorescence intensity per cell was determined by measuring about 500 randomly selected cells per sample per experiment.

*5,5´,6,6´-Tetrachloro-1,1´,3,3´-tetraethylbenzimi-dazolylcarbocyanine iodide (JC-1).* We used JC-1, a membrane potential–sensitive fluorescent probe (Life Technologies), to determine the mitochondrial membrane potential. JC-1 accumulates as orange aggregates in the mitochondria of cells with normal mitochondrial function and membrane potential and as green monomers in the mitochondria of cells with impaired mitochondrial function and membrane potential.

*RNA purification and reverse-transcription polymerase chain reaction (PCR).* Exponentially growing parental and ρ^0^ SAE cells were treated with 1 µg/cm^2^ asbestos for 12, 24, or 48 hr. Total cellular RNA was purified with the Ambion® mirVana™ miRNA Isolation Kits (Life Technologies). RNA concentration was measured with a NanoDrop 3300 fluorospectrometer (Thermo Fisher Scientific, Waltham, MA), and RNA quality was checked with an Agilent microfluidic RNA 6000 Nano Chip kit on the 2100 Bioanalyzer (Agilent Technologies, Santa Clara, CA). cDNA was generated by reverse transcription using High Capacity cDNA Reverse Transcription Kits [Applied Biosystems (Life Technologies)]. The reaction was carried out in 60 µL 1× buffer containing 10 ng/µL RNA template. The reverse-transcription-PCR conditions were 25°C for 10 min followed by 37°C for 120 min.

*Gene expression by TaqMan low-density array.* The mRNA expression level of 93 inflammation-related and 93 immune-responsive genes (178 in total, with eight overlapping genes) was assayed with Applied Biosystems’ TaqMan Gene Signature human immune and inflammation arrays on the 7900HT Fast Real-Time PCR System controlled by SDS software, version 2.3 [Applied Biosystems (Life Technologies)]. For each sample, a mixture of TaqMan Gene Expression Master Mix and cDNA equivalent to 250 ng total RNA was loaded onto the array and subjected to the amplification of 93 immune- or inflammation-related genes plus three endogenous controls.

*Statistical analysis.* Gene expression results were analyzed using RealTime StatMiner, version 4.1 (Integromics Inc., Granada, Spain). The relative quantity of each gene in the asbestos-treated group compared with the corresponding gene in the control group was calculated for all samples using 18S rRNA (ribosomal RNA) and GAPDH (glyceraldehyde 3-phosphate dehydrogenase) as endogenous controls. Gene clustering using single linkage and Pearson correlation was performed to visualize differences in gene expression. Network generation was carried out using Ingenuity Pathway Analysis (IPA) software, version 6.0 (Ingenuity Systems Inc., Redwood, CA). The correlation between asbestos doses and MN yields was examined using the Pearson correlation test. All other results were analyzed using one-way analysis of variance followed by Dunnett’s multiple comparison test. *p*-Values < 0.05 were considered statistically significant.

## Results

**Figure 1:**
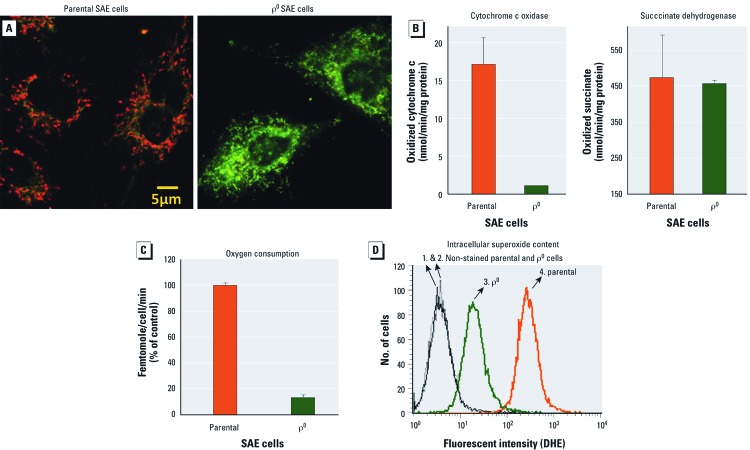
Verification of ρ^0^ status. (*A*) ρ^0^ SAE cells showed a loss of mitochondrial membrane potential as indicated by JC-1 staining. JC-1 formed orange aggregates in parental SAE cells but remained as green monomers in ρ^0^ SAE cells. (*B*) Compared with parental cells, ρ^0^ SAE cells showed a significant decrease in COX activity [an enzyme complex (IV) encoded jointly by mtDNA and nuclear DNA] but showed no change in succinate dehydrogenase activity (an enzyme complex encoded entirely by nuclear DNA). (*C*) ρ^0^ SAE cells have a significantly reduced oxygen consumption rate compared with the parental cells. (*D*) Intracellular superoxide content is indicated by dihydroethidium (DHE) fluorescence. ρ^0^ SAE cells (3) showed a decreased level compared with parental SAE cells (4). Each of the experiments shown in *A*–*D* was repeated three times.

*Characterization of parental and* ρ*^0^ SAE cells and cellular toxicity of asbestos.* Real-time PCR results showed that after 2 months of 50 ng/mL EtBr treatment to deplete mtDNA, the ratio of mtDNA to nuclear DNA in ρ^0^ SAE cells was 0.2 ± 0.08% of the ratio in parental SAE cells [see Supplemental Material, Table 1 (http://dx.doi.org/10.1289/ehp.1104287)]. Because mtDNA-encoded proteins are important components of mitochondrial respiration, we further examined mitochondrial functional markers in ρ^0^ SAE cells. The ρ^0^ status was confirmed by a loss of mitochondrial membrane potential gradient (Figure 1A), significantly decreased COX activity (Figure 1B), and oxygen consumption rate (Figure 1C), as well as a decrease in intracellular superoxide content (Figure 1D). All the experiments in the present study used ρ^0^ SAE cells within five passages to guarantee a true ρ^0^ status (see Supplemental Material, Table 1). For details regarding cell and mitochondria morphology, growth curves, and results of asbestos toxicity assays for both cell lines, see Supplemental Material, Figure 1.

**Figure 2:**
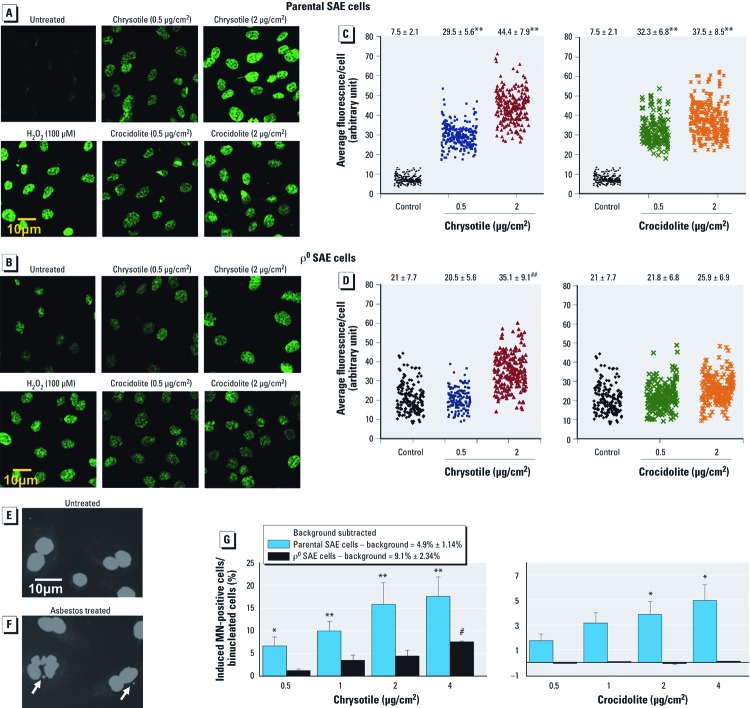
ρ^0^ SAE cells were significantly less sensitive than parental SAE cells to asbestos-induced 8-OHdG and MN formation. (*A*–*D*) Detection of 8-OHdG by immunofluorescence. (*A*,*B*) Representative images from untreated and treated (0.5 and 2 µg/cm^2^ for 48 hr; H_2_O_2_ treatment, 100 µM for 30 min) samples: parental cells (*A*) and ρ^0^ SAE cells (*B*). (*C,D*) Results of chrysotile and crocidolite treatments plotted with a horizontal jitter; each dot represents a single cell quantified. The mean ± SD fluorescence intensity per cell shown at the top was obtained from approximately 200 cells per sample. H_2_O_2_-treated group, 35.8 ± 6.52 (not plotted here). (*E,F*) Untreated (*E*) and 2-µg/cm^2^ chrysotile–treated (*F*) SAE cells. Arrows indicate MN. (*G*) Asbestos-induced MN-positive cells, background subtracted (mean ± SD). Pooled data were obtained from three independent experiments. Chrysotile (left) induced MN in both parental and ρ^0^ SAE cells in a dose-dependent manner (*r* = 0.89 and 0.9, respectively, Pearson correlation). Crocidolite (right) induced a dose-dependent increase (*r* = 0.97, Pearson correlation) in the frequency of MN-positive cells in parental cells but had no effect on ρ^0^ SAE cells. *p < 0.05, and **p < 0.01, Dunnett’s test compared with untreated SAE cells. ^#^*p* < 0.05, and ^# #^*p* < 0.01, Dunnett’s test compared with untreated ρ^0^ SAE cells.

*Asbestos fibers induced nuclear DNA oxidative damage.* Initially, we examined the role of mitochondria in asbestos-induced oxidative damage of the nuclear genome. To do this, both parental and ρ^0^ SAE cells were subjected to various doses of chrysotile and crocidolite exposure for 48 hr. The formation of 8-OHdG, a DNA lesion known to form upon oxidative damage, was measured by immunofluorescence staining. Importantly, the 8-OHdG staining colocalized with nucleus staining by PI (data not shown). A higher baseline 8-OHdG level was observed in untreated ρ^0^ SAE cells (20.96 ± 7.72; Figure 2B,D) compared with the parental SAE cells (7.45 ± 2.05; Figure 2A,C). However, asbestos-induced 8-OHdG levels were much lower in ρ^0^ SAE cells (Figure 2D) than in parental SAE cells (Figure 2C; *p* < 0.01, Dunnett’s test). In addition, at a dose of 2 µg/cm^2^, chrysotile induced a higher level of 8-OHdG than crocidolite in both parental and ρ^0^ SAE cells (*p* < 0.05, Dunnett’s test; Figure 2C,D).

*Asbestos fibers induced chromosomal breaks.* Asbestos is a well-established chromosomal mutagen that induces multilocus deletions and MN (Jaurand 1997). We determined the asbestos-induced MN yield in both parental and ρ^0^ SAE cells to confirm the role of mitochondria in asbestos-mediated chromosomal damage. MN were scored on binucleated ρ^0^ or parental SAE cells after 48 hr of asbestos exposure (Fenech 2000). Representative photomicrographs indicate MN formation in 2 µg/cm^2^ chrysotile–treated SAE cells (Figure 2F) but not untreated controls (Figure 2E). The results show that ρ^0^ SAE cells had significantly higher background MN (9.1 ± 2.34%) than did parental SAE cells (4.9 ± 1.14%; *p* < 0.05, Dunnett’s test). Asbestos treatment induced significant increases in MN yield in parental SAE cells (Figure 2G). The frequency of MN-positive SAE cells increased in a dose-dependent manner, from 6.7 ± 1.9% after 0.5 µg/cm^2^ to 17.6 ± 4.3% after 4 µg/cm^2^ chrysotile exposure (*r* = 0.89, Pearson correlation), and from 1.7 ± 0.5% after 0.5 µg/cm^2^ to 5 ± 1.2% after 4 µg/cm^2^ crocidolite exposure (*r* = 0.9, Pearson correlation). Although chrysotile induced a slight dose-dependent (*r* = 0.97, Pearson correlation) increase in MN yield among ρ^0^ SAE cells (range, 1.2 ± 0.4% to 7.6 ± 0.2%), MN yield was not affected by crocidolite treatment at any dose examined (Figure 2G). These results show that mitochondria-dysfunctional ρ^0^ SAE cells were significantly less sensitive to chrysotile- and crocidolite-induced MN formation than were parental SAE cells (*p* < 0.01, parental vs. ρ^0^ SAE cells for each of the doses examined, Dunnett’s test). Chrysotile fibers were generally more potent than crocidolite in inducing MN in both cell lines (*p* < 0.01, chrysotile vs. crocidolite for each of the doses examined, Dunnett’s test).

**Figure 3:**
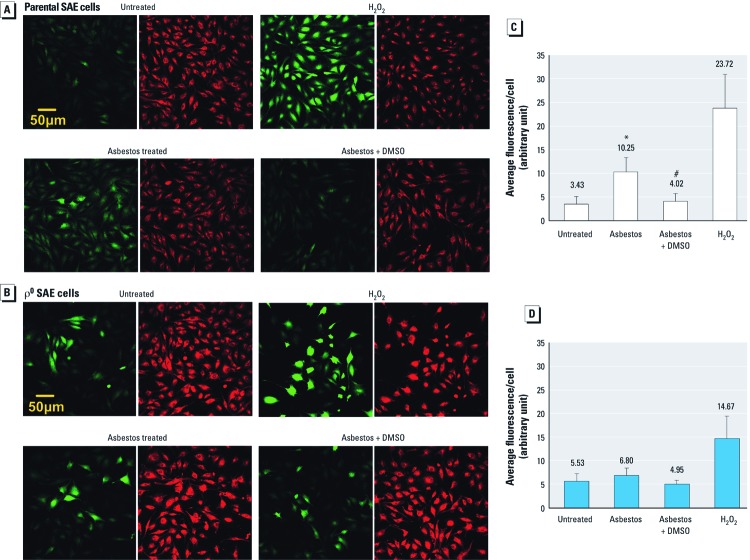
Asbestos-induced intracellular ROS in parental but not ρ^0^ SAE cells. (*A,B*) Representative photomicrographs of parental (*A*) and ρ^0^ SAE cells (*B*): Untreated, controls; asbestos treated, 0.5 µg/cm^2^ chrysotile for 24 hr; asbestos + DMSO, 0.5% DMSO for 24 hr, followed by 0.5 µg/cm^2^ chrysotile plus 0.5% DMSO for 24 hr; H_2_O_2_, 0.2 mM H_2_O_2_ for 30 min. CM-DCF fluorescence (green) indicates intracellular ROS level; red indicates Cell Tracker Red counterstaining. (*C*,*D*) Relative quantification of CM-DCF fluorescence: parental (*C*) and ρ^0^ SAE cells (*D*). The green fluorescence was normalized to the Cell Tracker staining to account for the difference in cell thickness between the parental and ρ^0^ SAE cells. The mean ± SD fluorescence per cell was obtained from three independent experiments. *p < 0.05; Dunnett’s test compared with untreated SAE cells; ^#^*p* < 0.05, Dunnett’s test compared with asbestos-treated SAE cells.

*Increase of intracellular oxidants in asbestos-treated SAE cells.* To verify the contribution of mitochondria-derived ROS to asbestos-induced nuclear oxidative damage and lesions, we compared asbestos-induced intracellular oxidant production in ρ^0^ versus parental SAE cells. The overall ROS level was determined using CM-H_2_DCFDA, a cellular membrane–permeable nonfluorescent probe that can be irreversibly oxidized by intracellular ROS into a green fluorescent product, CM-DCF. In SAE cells, 24-hr asbestos treatment tripled CM-DCF fluorescence level over background (*p* < 0.05, Dunnett’s test; Figure 3A,C). Pretreating and co-treating cells with the radical scavenger DMSO prevented induction of oxyradicals by asbestos, as indicated by the decrease in average fluorescence per cell (Figure 3A,C; *p* < 0.05, Dunnett’s test). In contrast, although the baseline ROS level was higher in ρ^0^ SAE cells than in parental SAE cells, treatment with asbestos induced little or no increase in intracellular oxidants (Figure 3B,D). DMSO treatment slightly reduced the fluorescence level in asbestos-exposed ρ^0^ SAE cells with no statistical significance (Figure 3B,D). Notably, H_2_O_2_ induced an abundant increase of intracellular ROS in both parental and ρ^0^ SAE cells (Figure 3). This clearly indicates that H_2_O_2_ induced ROS via a non-mitochondrial–related mechanism, in contrast with asbestos.

**Figure 4:**
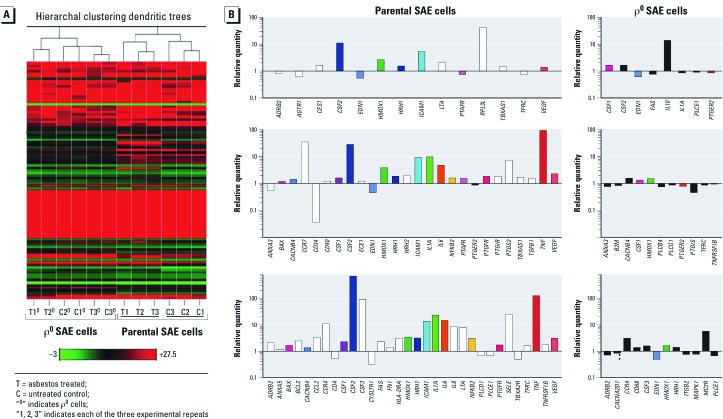
Asbestos treatment induced an increase in the expression of immune- and inflammation-related genes in parental but not ρ^0^ SAE cells. (*A*) Heatmap depicting the expressions of all genes measured by low-density array. The gene expression levels for each sample were mean centered and hierarchically clustered according to similarity in overall gene expression pattern. The plotted data represent results from three independent biological repetitions. The color scale bar indicates the fold decrease (green) or increase (red) in gene expression compared with the mean expression level (black) of all genes examined. (*B*) A list of genes that contributed to the clustering in *A*, that is, genes significantly induced or suppressed by asbestos (differentially expressed in response to 1-µg/cm^2^ asbestos treatment). Columns represent the relative quantity of asbestos-induced differentially expressed genes at 12, 24, and 48 hr for both parental (left) and ρ^0^ (right) SAE cells. Genes consistently differentially expressed across time points appear in the same colors. White (parental cells) and black (ρ^0^ SAE cells) columns indicate genes that were differentially expressed at one time point only. Data were obtained from three biological repetitions. A detailed list of all differentially expressed genes is available in Supplemental Material, Table 3 (SAE cells) and Table 4 (ρ^0^ SAE cells) (http://dx.doi.org/10.1289/ehp.1104287).

*Gene expression and clustering analysis of all genes examined.* The above experiments indicated that mitochondria-derived ROS were a major factor mediating direct nuclear damage in asbestos-treated SAE cells. Next, we wanted to determine whether mitochondria-derived ROS could also induce relevant nuclear signaling pathways. We analyzed the mRNA expression of 178 genes [see Supplemental Material, Table 2 (http://dx.doi.org/10.1289/ehp.1104287)] involved in immune and inflammation pathways by TaqMan low-density arrays. The expression of these genes was examined at 12, 24, and 48 hr in four experimental groups: *a*) untreated SAE cells, *b*) 1-µg/cm^2^ asbestos-treated SAE cells, *c*) untreated ρ^0^ SAE cells, and *d*) 1-µg/cm^2^ asbestos-treated ρ^0^ SAE cells. Gene clustering analysis was used to compare the global gene expression patterns among different samples. It was performed on raw data from three independent biological repetitions. The result shows that SAE cells clustered by treatment, an indication that asbestos had a substantial effect on gene expression (Figure 4A, right). In contrast, asbestos treatment did not affect gene expression in ρ^0^ SAE cells to the same extent, and samples are poorly clustered (Figure 4A, left).

*Differentially expressed genes in asbestos-treated groups versus controls.* Genes differentially expressed (significantly induced or suppressed by asbestos, paired *t*-test, *p* < 0.05) in asbestos-treated groups versus untreated controls were identified for parental and ρ^0^ SAE cells, respectively. The relative quantity for each of the differentially expressed genes at 12, 24, and 48 hr is plotted in Figure 4B. A detailed list of all of the differentially expressed genes in SAE cells is provided in Supplemental Material, Table 3 (http://dx.doi.org/10.1289/ehp.1104287). Asbestos induced a time-dependent increase in the expression of a subset of immune- and inflammation-related genes in SAE cells (Figure 4B, left). Although 12-hr treatment affected the expression of only a few genes, with 24- and 48-hr treatments, the expression of 26 and 32 genes, respectively, was significantly altered (mostly up-regulated; paired *t*-test, *p* < 0.05) by asbestos. About half the genes differentially expressed at 24 hr were consistently up-regulated at 48 hr in asbestos-treated SAE cells (Figure 4B, left; see also Supplemental Material, Table 3). In contrast, asbestos had little effect on gene expression in ρ^0^ SAE cells (Figure 4B, right). Six up-regulated genes (paired *t*-test, *p* < 0.05) were identified at 48 hr, but the fold changes were all < 2 except for *MC2R* (melanocortin 2 receptor; ratio, 5.66) and *CCR4* (chemokine receptor 4; ratio, 3.24; Figure 4B, right; see also Supplemental Material, Table 4).

It should be noted that the baseline expression of some genes in ρ^0^ SAE cells differed from that in parental SAE cells [Supplemental Material, Table 5 (http://dx.doi.org/10.1289/ehp.1104287)]. Because the background intracellular oxidant level is increased in ρ^0^ SAE cells (Figure 3B,D), it is necessary to examine whether the baseline gene expression pattern in untreated ρ^0^ SAE cells is similar to that in asbestos-treated SAE cells where oxidative stress is induced (Figure 3A,C). Importantly, our data did not reveal such a similarity. This suggests that the failure of asbestos to induce any big change in gene expression in ρ^0^ SAE cells was not due to the preactivation of these genes during the process of ρ^0^ SAE cell generation, but was most likely a consequence of the lack of mtDNA and mitochondrial function.

**Figure 5:**
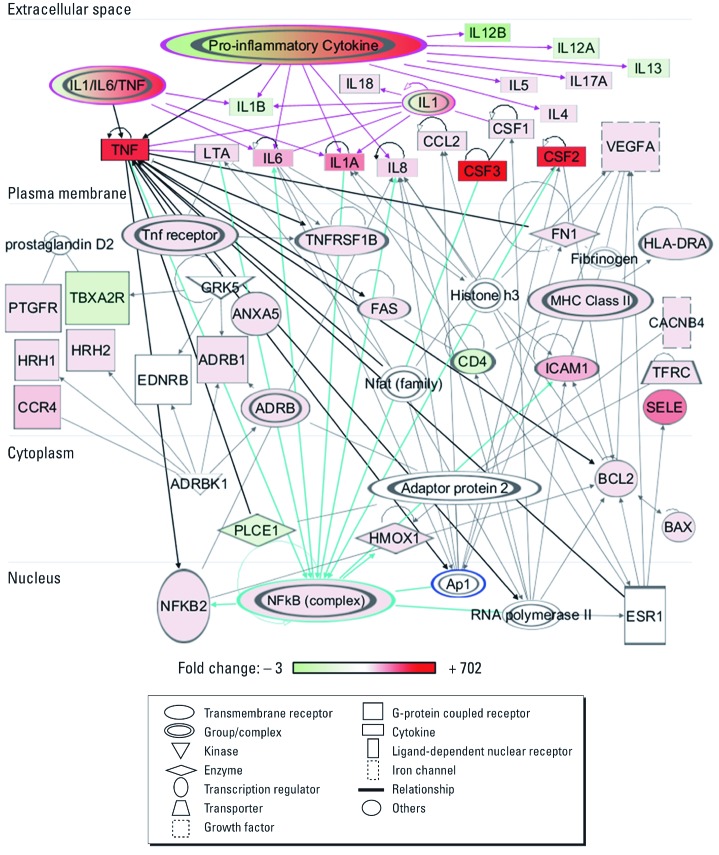
Network of genes mediated by asbestos after 48 hr of treatment in SAE cells. The color scale indicates the fold change in gene expression level for the 32 genes: green, down-regulation; red, up-regulation; uncolored molecules, expression levels were not examined in the present study.

*Network analysis.* We observed significant induction of 27 genes and down-regulation of 5 genes in SAE cells 48 hr after asbestos treatment [Figure 4B; see also Supplemental Material, Table 3 (http://dx.doi.org/10.1289/ehp.1104287)]. Gene ontology–related pathway analysis using IPA software showed that these 32 genes were significantly associated with induction of oxidative stress, xenobiotic metabolic processes, proapoptosis, and other processes (Supplemental Material, Table 6). Furthermore, a group of highly interconnected networks of the 32 asbestos-responsive genes in SAE cells was constructed by IPA (Figure 5), based on direct interactions stored in the Ingenuity Knowledge Base (Ingenuity Systems Inc., Redwood City, CA) a collection of experimentally confirmed relationships between molecules. The networks demonstrate that the 32 genes were directly related to activation of transcription factors nuclear factor κB (NF-κB) and activator protein-1 (AP-1) signaling pathways, and to downstream signaling including various interleukins (ILs), tumor necrosis factor (TNF) and TNF receptor (TNFR), colony stimulating factors 1–3 (CSFs), and other pro-inflammatory cytokines or growth factors (Figure 5). These signaling pathways are known to be oxidative stress–responsive and have been consistently reported to be activated by asbestos exposure (Manning et al. 2002; Martindale and Holbrook 2002). Network analysis of significant genes from the 24-hr group of SAE cells show similar results (data not shown). By contrast, IPA revealed that the 12 genes differentially expressed in ρ^0^ SAE cells at 48 hr after asbestos treatment (Figure 4; see also Supplemental Material, Table 4) were associated with an inhibition of mitogen-activated protein kinase, AP-1/JUN, and TNF signaling cascades (Supplemental Material, Figure 2).

## Discussion

The biological effects of oxidative stress in many disease states manifest as both direct damage of macromolecules and modification of redox signaling pathways in target cells (Valko et al. 2007). In the present study, both effects were detected in parental human SAE cells, whereas minimal effects were observed in mitochondria-dysfunctional ρ^0^ human SAE cells. These results collectively provide direct evidence that mitochondria may play an essential role in regulating asbestos-induced genotoxicity and cell signaling alterations. These processes are most likely mediated through mitochondria-originated ROS. To our knowledge, this is the first report of the direct involvement of mitochondria in asbestos genotoxicity and pathogenicity in relevant human target cells.

Consistent with our previous findings on the role of oxyradicals and extranuclear targets in fiber mutagenesis in A_L_ cells (Xu et al. 1999, 2002, 2007), the present study found asbestos fiber treatment of mitochondria-dysfunctional ρ^0^ human SAE cells, which consume 10-fold less oxygen than parental SAE cells, caused significantly lower intracellular oxidant production, nuclear oxidative damage (i.e., 8-OHdG), and MN induction than observed in asbestos-treated parental SAE cells. These data strongly suggest that mitochondria-associated oxidants are a main contributor to asbestos-induced intracellular oxidative stress, and that reductions in asbestos-induced oxidative and chromosomal damage in ρ^0^ SAE cells compared with parental SAE cells were due to the lack of mitochondria-derived ROS. The oxidation of CM-H_2_DCFDA by reactive radical species, as detected using confocal microscopy, provided strong evidence that asbestos induced ROS and reactive nitrogen species that could be inhibited by DMSO (Xu et al. 2007).

Reported human asbestos exposure levels vary depending on the sources and types of exposure (Dodson et al. 1997; Roggli et al. 2004). Asbestos concentrations used in this study ranged from 0.5 to 4 µg/cm^2^, well below the reported dose (10 µg/cm^2^) that can cause overload, i.e., a retained burden of asbestos fibers resulting from an impairment of the asbestos clearance capacity, for cultured cells (Faux et al. 2003; International Life Sciences Institute 2000). Although crocidolite has been established by numerous studies to be more carcinogenic than chrysotile in humans (Berman and Crump 2008; Hodgson and Darnton 2000), in the present study chrysotile was found to be more DNA damaging than crocidolite fibers. Our results show that chrysotile, but not crocidolite, can induce a significant increase in 8-OHdG and MN formation in ρ^0^ SAE cells at 2 and 4 µg/cm^2^, respectively. It is possible that the chemical and physical properties of the material used in our study were modified during fiber processing and may vary from other batches. Furthermore, our findings suggest that in addition to ROS production, chrysotile might induce nuclear mutation through other mechanisms.

Notably, baseline levels of 8-OHdG, MN, and intracellular oxidants in ρ^0^ SAE cells were higher than corresponding background levels in parental SAE cells. MN may have been increased because of EtBr treatment (McCann et al. 1975), although the mutagenic effect of EtBr in mammalian systems remains unclear (National Toxicology Program 2005). The need for extended EtBr treatment is a potential drawback of the ρ^0^ SAE cell model; nevertheless, growth kinetics in ρ^0^ SAE cells are similar to those in wild-type cells. Until the ideal mitochondria-dysfunctional cell model is generated, ρ^0^ SAE cells represent the next best alternative model available. EtBr is a known selective inhibitor of mitochondrial transcription and replication processes that has no effect on nuclear DNA replication at the concentration used in the study (Leibowitz 1971; Seidel-Rogol and Shadel 2002). EtBr has effectively been used to reduce the copy number of mtDNA in proliferating cells (King and Attardi 1996). The effect of EtBr is reversible, so that once EtBr is removed from the culture medium, cells are able to repopulate their mtDNA (King and Attardi 1989). As such, the present study used ρ^0^ SAE cells within five passages to guarantee a true ρ^0^ status. The oxidants in untreated ρ^0^ SAE cells may have been derived from sources other than the mitochondrial respiration chain because the baseline intracellular superoxide content in ρ^0^ SAE cells was low compared with parental SAE cells. This unidentified source of oxidants may also have contributed to the substantially increased background levels of 8-OHdG in ρ^0^ SAE cells. It is possible that in ρ^0^ SAE cells the DNA repair capacity is decreased or the antioxidant enzymes are impaired because similar results were observed in the nucleus of ρ^0^ HeLa cells (Delsite et al. 2003). In the near future, we will try to use genetic tools to block mitochondrial ROS production and identify the other sources of ROS production in ρ^0^ SAE cells.

Using gene arrays and pathway analyses, we showed that mitochondria-associated oxidants were able to mediate redox signaling in response to asbestos exposure. Chrysotile asbestos induced a significant increase in the expression of immune- and inflammation-related genes in normal SAE cells but had minimal effect on gene expression in ρ^0^ SAE cells in terms of both the number of genes affected and the magnitude of up- or down-regulation observed in affected genes. Asbestos-responsive genes in parental SAE cells were significantly associated with activation of cellular signaling pathways, including NF-κB, AP-1, and pro-inflammatory cytokines, chemokines, and growth factors. These data provide additional support for our hypothesis that mtDNA and mitochondria-derived ROS are important in mediating asbestos-induced nuclear DNA and signaling alterations. Future gene knock-down studies in parental SAE cells or gene overexpression in ρ^0^ SAE cells may further illuminate the role of these signaling pathways in the asbestos-induced cellular response.

## Conclusion

The link between asbestos exposure and the development of lung cancer and mesothelioma in humans is unequivocal, but the underlying carcinogenic mechanism is not known. Using newly generated mtDNA-deficient (ρ^0^) human SAE cells, we showed that mitochondria are a major cytoplasmic target of asbestos. Our earlier studies using asbestos fiber–treated cytoplasts and fusion with nontreated karyoplasts provided circumstantial evidence of the role of mitochondria in fiber genotoxicity (Xu et al. 2007). Results of the present study expand on our earlier findings and suggest that mitochondria-associated oxidants mediate asbestos-associated nuclear damage and initiate redox-responsive signaling cascades in target cells. These data provide new insights into the molecular mechanisms of asbestos-induced genotoxic responses and provide a base for better prevention and treatment of fiber-mediated diseases.

## Supplemental Material

(3.1 MB) PDF
