# Supplementary material for: Mitochondria-Derived Reactive Intermediate Species Mediate Asbestos-Induced Genotoxicity and Oxidative Stress–Responsive Signaling Pathways
Source: Environ Health Perspect. 2012 Mar 7;120(6):840–7. doi: 10.1289/ehp.1104287 (PMC3385428; doi:10.1289/ehp.1104287)
Supplement: (3.1 MB) PDF [file ehp.1104287.s001.pdf]

Supplemental Material for Environmental Health Perspectives

Mitochondria-Derived Reactive Intermediate Species Mediate Asbestos-induced Genotoxicity and Oxidative Stress-Responsive Signaling Pathways

Sarah X.L. Huang[1], Michael A. Partridge[2 ‡], Shanaz A. Ghandhi[2], Mercy M. Davidson[3], Sally A. Amundson[2], and Tom K. Hei[1, 2, 3]*

[1]Department of Environmental Health Sciences, Mailman School of Public Health, Columbia University, New York, NY

[2]Center for Radiological Research, [3]Department of Radiation Oncology, College of Physicians & Surgeons, Columbia University, New York, NY

[‡]Current address: Regeneron Pharmaceuticals, Tarrytown, NY 10591

***Corresponding author:** Professor Tom K. Hei, Center for Radiological Research, College of Physicians and Surgeons, Columbia University, 630 West 168th Street, VC-11-205, New York, NY 10032. Phone: 212-305-8462; Fax: 212-305-3229; E-mail: tkh1@columbia.edu.

Supplemental Material

Methods 2

Toxicity of asbestos 2

Real-time Quantitative PCR 2

Mitochondrial membrane potential 2

Oxygen consumption 2

Cytochrome c Oxidase (*COX*) and succinate dehydrogenase (*SDH*) activity 3

Intracellular superoxide measurement with dihydroethidium (*DHE*) 3

Table 1. Real-time PCR screening of mtDNA content in EtBr-treated SAE cells and verification of the ρ^0 status. 4

Table 2. A list of all the genes examined. 5

Table 3. Differentially expressed genes in asbestos-treated SAE cells. 6

Table 4. Differentially expressed genes in asbestos-treated ρ^0 SAE cells. 7

Table 5. Differentially expressed genes in ρ^0 versus parental SAE cells. 8

Table 6. Ingenuity toxicity list of functions and pathways relevant to asbestos exposure. 9

Figure 1. Characterization of parental and ρ^0 SAE cells.. 10

Figure 2. Network of genes mediated by asbestos after 48 hr of treatment in ρ^0 SAE cells.. 11

References.. 12

Supplemental Material, Methods

Toxicity of asbestos

The cellular toxicity of asbestos was measured by determining the survival fraction with the CyQuant cell proliferation Assay kit (Invitrogen). Briefly, exponentially growing cells in a 96-well tissue culture plate were treated with graded doses of asbestos fibers (0.5, 1, 2, and 4 μg/cm^2) for 72 hours; then culture media were removed and the cellular nuclei acids content of each control and treated group was measured with a fluorescence excitation of 480nm using a Synergy 2 multi-detection Microplate Reader (BioTek Instruments, Inc, Vermont). The surviving fractions (percentage over untreated controls) for treated groups were calculated.

Real-time Quantitative PCR

The mtDNA copy number was determined by real-time SYBR Green PCR using the Applied Biosystems 7300 Real-time PCR System (Applied Biosystems). Genomic DNA was isolated and concentration was measured by spectrophotometry. For each sample, we amplified a 189-bp fragment of the nuclear encoded *18S* rRNA gene and a 172-bp fragment of the mtDNA encoded *12S* rRNA gene. The primer sequences are as follows: 18S sense, 5'-GGAGTATGGTTGCAAAGCTG-3'; 18S antisense, 5'-CGCTCCACCAACTAAGAACG-3'; 12S sense, 5'-AGAACACTACGAGCCACAGC-3'; and 12S antisense, 5'-ACTTGCGCTTACTTTGTAGCC-3'. All reactions were done in triplicate. The PCR conditions were: 95°C for 15 min followed by 40 cycles at 95°C for 30 s, 55°C for 30 s, and 72°C for 30 s. Relative quantification of mtDNA reported as mtDNA/nDNA ratio was done with the comparative threshold cycle (C_T) method as described previously (Partridge *et al.* 2007; Shao *et al.* 2006).

Mitochondrial membrane potential

JC-1, a membrane potential–sensitive fluorescent probe (Invitrogen), was used to determine the mitochondrial membrane potential of ρ^0 SAE cells (Liu et al. 2005; Reers et al. 1995). Exponentially growing parental and ρ^0 SAE cells were treated with 10 μmol/L JC-1 in growth medium for 30 min at 37°C. Extra dye was removed by washing with warm PBS and cells were maintained in regular medium on a heated 37°C stage. Cells were immediately visualized and images were captured on a Nikon laser confocal microscope (Nikon eclipse TE2000-U; excitation 488 nm and 543 nm for green and orange fluorescence, respectively).

Oxygen consumption

The oxygen consumption rate in live cells was measured as described previously (King et al. 1992; Partridge et al. 2007). Cells were counted and a minimum of 5 x 10^6 cells were resuspended in 1.5 mL of Opti-MEM (glucose free and sodium pyruvate supplemented

medium). Oxygen concentration was monitored over 3 min and recorded for every 10 seconds at 37°C in a Hansatech (MA) Clark's oxygen electrode unit.

Cytochrome c Oxidase (COX) and succinate dehydrogenase (SDH) activity

COX and SDH activities were measured with biochemical assays as described (King et al. 1967; Partridge et al. 2007; Salviati et al. 2002). COX activity was indicated by the capability of the cell lysates to oxidize reduced cytochrome *c* at 550 nm (nmol oxidized cytochrome *c* per min per ml cell lysate) as measured by spectrophotometry. SDH activity was assessed by measuring the oxidization of succinate coupled to the reduction of DCPIP (2,6-dichlorophenolindophenol, an electron acceptor that is blue when oxidized and colorless when reduced) by the SDH reaction at 600 nm with spectrophotometry. The enzyme activity was indicated by nmol oxidized succinate per min per ml cell lysate. Both COX and SDH activities were normalized to mg of protein in the cell lysate.

Intracellular superoxide measurement with dihydroethidium (DHE)

Intracellular superoxide was determined using the fluorescent probe DHE (Invitrogen) as described previously (Zhou et al. 2008). Exponentially growing ρ^0 or parental SAE cells were stained with 2µmol/L DHE in regular medium for 45 min at 37°C. Cells were then trypsinized and suspended in PBS. The DHE fluorescence was measured by flow cytometry in the FL3 channel on a FACSCalibur (Becton Dickinson).

Supplemental Material, Table 1. Real-time PCR screening of mtDNA content in EtBr-treated SAE cells and verification of the ρ^0 status.

**Samples**		**mtDNA/nDNA* Ratio**
SAE Cells		1.0000 ± 0.0446
EtBr-treated SAE cells	23 days	0.0100 ± 0.0037
	29 days	0.0059 ± 0.0013
	37 days	0.0039 ± 0.0005
	55 days	0.0027 ± 0.0005
	63 days	0.0022 ± 0.0008
ρ^0 SAE cells +1 passage		0.0059 ± 0.0004
ρ^0 SAE cells +3 passage		0.0097 ± 0.0024
ρ^0 SAE cells +5 passage		0.0165 ± 0.0019
ρ^0 SAE cells +7 passage		0.0779 ± 0.0275
ρ^+ primary human skin Fibroblast		1.0000 ± 0.1877
ρ^0 primary human skin Fibroblast		0.0046 ± 0.0007

* nDNA = nuclear DNA

Note: mitochondrial DNA contents relative to nuclear DNA contents was determined using quantitative real-time PCR. The resultant mtDNA/ n DNA ratios in EtBr-treated samples were normalized to the ratio in untreated SAE cells.

Supplemental Material, Table 2. A list of all the genes examined.

A2M	*CCR5*	*FN1*	*IL2*	*MAPK3*	*PTGER2*
ACE	*CCR7*	*GNLY*	*IL2RA*	*MAPK8*	*PTGER3*
ADRB1	*CD19*	*GUSB*	*IL2RB*	*MC2R*	*PTGFR*
ADRB2	*CD28*	*GZMB*	*IL2RG*	*MYH6*	*PTGIR*
AGTR1	*CD34*	*HLA-DRA*	*IL3*	*NFKB1*	*PTGIS*
AGTR2	*CD38*	*HLA-DRB1*	*IL4*	*NFKB2*	*PTGS1*
ALOX12	*CD3E*	*HMOX1*	*IL5*	*NOS2A*	*PTGS2*
ALOX5	*CD4*	*HPGD*	*IL6*	*NR3C1*	*PTPRC*
ANXA1	*CD40*	*HRH1*	*IL7*	*PDE4A*	*REN*
ANXA3	*CD40LG*	*HRH2*	*IL8*	*PDE4B*	*RPL3L*
ANXA5	*CD68*	*HRH3*	*IL9*	*PDE4C*	*SELE*
B2M	*CD80*	*HTR3A*	*ITGAL*	*PDE4D*	*SELP*
BAX	*CD86*	*HTR3B*	*ITGAM*	*PGK1*	*SKI*
BCL2	*CD8A*	*ICAM1*	*ITGB1*	*PLA2G10*	*SMAD3*
BCL2L1	*CES1*	*ICOS*	*ITGB2*	*PLA2G1B*	*SMAD7*
BDKRB1	*COL4A5*	*IFNG*	*KLK1*	*PLA2G2A*	*STAT3*
BDKRB2	*CSF1*	*IKBKB*	*KLK14*	*PLA2G2D*	*TBX21*
C3	*CSF2*	*IL10*	*KLK15*	*PLA2G4C*	*TBXA2R*
CACNA1C	*CSF3*	*IL12A*	*KLK2*	*PLA2G5*	*TBXAS1*
CACNA1D	*CTLA4*	*IL12B*	*KLK3*	*PLA2G7*	*TFRC*
CACNA2D1	*CXCL10*	*IL13*	*KLKB1*	*PLCB2*	*TGFB1*
CACNB2	*CXCL11*	*IL15*	*KNG1*	*PLCB3*	*TNF*
CACNB4	*CXCR3*	*IL17*	*LRP2*	*PLCB4*	*TNFRSF18*
CASP1	*CYP1A2*	*IL18*	*LTA*	*PLCD1*	*TNFRSF1A*
CCL19	*CYP7A1*	*IL1A*	*LTA4H*	*PLCE1*	*TNFRSF1B*
CCL2	*CYSLTR1*	*IL1B*	*LTB4R*	*PLCG1*	*TNFSF13B*
CCL3	*ECE1*	*IL1R1*	*LTB4R2*	*PLCG2*	*VCAM1*
CCL5	*EDN1*	*IL1R2*	*LTC4S*	*PRF1*	*VEGF*
CCR2	*FAS*	*IL1RAPL2*	*MAPK1*	*PTAFR*	
CCR4	*FASLG*	*IL1RL1*	*MAPK14*	*PTGDR*	

Note: 8 genes from the pre-designed immune and inflammation arrays overlapped with each other. 178 genes are in list, excluding 3 housekeeping genes in each array,

Supplemental Material, Table 3. Differentially expressed genes in asbestos-treated SAE cells.

SAE cells							
	**Genes**	**RQ (Asb vs. Ctrl*)**	**P.Value†**		**Genes**	**RQ (Asb vs. Ctrl)**	**P.Value†**
12 hr	*ADRB2*	0.85	0.032	48hr	*ADRB1*	2.13	0.01
	AGTR1	0.61	0.024		*ANXA5*	1.15	0.008
	CES1	1.67	0.012		*BAX*	1.69	0.038
	CSF2	11.47	0.049		*BCL2*	2.38	0.013
	EDN1	0.53	0.033		*CACNB4*	1.37	0.004
	HMOX1	2.73	0.007		*CCL2*	3.33	0.027
	HRH1	1.53	0.021		*CCR4*	10.82	0.019
	ICAM1	5.61	0.037		*CD4*	0.54	0.008
	LTA	2.16	0.037		*CSF1*	2.35	0.032
	PTAFR	0.74	0.002		*CSF2*	702.65	0.008
	RPL3L	42.57	0.008		*CSF3*	92.48	0.019
	TBXAS1	1.53	0.034		*CYSLTR1*	0.32	0.001
	TFRC	0.77	0.044		*FAS*	2.21	0.034
	VEGF	1.44	0.003		*FN1*	1.41	0.014
24 hr	*ANXA3*	0.55	0.033		*HLA-DRA*	3.00	0.009
	BAX	1.22	0.001		*HMOX1*	3.46	0.014
	CACNB4	1.42	0.025		*HRH1*	3.12	0.017
	CCR7	35.71	0.00002		*ICAM1*	13.53	0.007
	CD34	0.04	0.006		*IL1A*	23.60	0.049
	CD40	1.23	0.043		*IL6*	14.96	0.013
	CSF1	1.66	0.008		*IL8*	8.69	0.008
	CSF2	29.70	0.007		*LTA*	7.93	0.018
	ECE1	1.21	0.006		*NFKB2*	3.19	0.014
	EDN1	0.46	0.012		*PLCD1*	0.70	0.027
	HMOX1	4.01	0.006		*PLCE1*	0.67	0.017
	HRH1	1.87	0.032		*PTGFR*	1.71	0.037
	HRH2	1.95	0.028		*SELE*	24.53	0.003
	ICAM1	9.34	0.006		*TBXA2R*	0.52	0.047
	IL1A	9.71	0.027		*TFRC*	1.61	0.008
	IL6	4.97	0.03		*TNF*	130.57	0.001
	NFKB2	1.68	0.049		*TNFRSF1B*	1.77	0.025
	PTAFR	1.59	0.045		*VEGF*	3.16	0.013
	PTGER2	0.91	0.044				
	PTGFR	1.89	0.032				
	PTGIR	1.80	0.0004				
	PTGS2	7.33	0.002				
	TBXAS1	1.71	0.026				
	TGFB1	1.58	0.028				
	TNF	96.79	0.006				
	VEGF	2.30	0.006				

*Asb =Asbestos, Ctrl = Control;
†Paired T-test by Statminer
The RQ value of each gene is plotted in Main text, Figure 4B, *left*.

Supplemental Material, Table 4. Differentially expressed genes in asbestos-treated ρ^0 SAE cells.

	ρ^0 SAE cells						
	**Genes**	**RQ (Asb vs. Ctrl*)**	**P.Value†**		**Genes**	**RQ (Asb vs. Ctrl)**	**P.Value†**
**12 hr**	*CSF1*	1.17	0.011	**48hr**	*ADRB2*	0.70	0.017
	CSF2	1.91	0.005		*CACNA2D1*	0.80	0.028
	EDN1	0.60	0.047		*CCR4*	3.24	0.039
	FAS	0.76	0.016		*CD68*	1.38	0.03
	IL10	13.79	0.004		*CSF3*	1.64	0.044
	IL1A	0.83	0.024		*EDN1*	0.50	0.042
	PLCE1	0.90	0.015		*HMOX1*	1.60	0.031
	PTGER2	0.85	0.012		*HRH1*	1.43	0.039
**24 hr**	*ANXA3*	0.74	0.0001		*ITGB2*	0.74	0.018
	B2M	0.81	0.025		*MAPK1*	0.75	0.018
	CACNB4	1.59	0.018		*MC2R*	5.66	0.042
	CSF1	1.32	0.038		*PLCE1*	0.64	0.044
	HMOX1	1.50	0.012				
	PLCB4	0.71	0.014				
	PLCG1	0.87	0.046				
	PTGER2	0.76	0.036				
	PTGIS	0.45	0.003				
	TFRC	0.85	0.008				
	TNFRSF1B	0.77	0.024				

*Asb =Asbestos, Ctrl = Control;
†Paired T-test by StatMiner
The log_{10}RQ value of each gene is plotted in Main text, Figure 4B, *right*.

Supplemental Material, Table 5. Expressed genes at baseline in ρ^0 versus parental SAE cells.

**Genes**	**RQ (ρ^0 vs. parental SAE cells)**	**P.Value***	**Genes**	**RQ (ρ^0 vs. parental SAE cells)**	**P.Value***
ACE	0.1	0.006	*IL1B*	100.5	0.037
ADRB1	3.4	0.003	*IL1RAPL2*	2.8	0.006
ANXA3	8.3	0.004	*IL6*	3.4	0.005
ANXA5	0.5	0.041	*IL7*	1.7	0.038
C3	3.2	0.009	*IL8*	0.4	0.032
CACNA2D1	7.6	0.047	*ITGB2*	0.5	0.02
CCL2	0.2	0.017	*KLKB1*	0.3	0.032
CCL3	282.2	0.006	*MAPK1*	0.6	0.004
CCL5	46.8	0.003	*MC2R*	0.6	0.031
CD40	0.5	0.009	*NFKB1*	0.6	0.044
CES1	13.1	0.013	*PDE4A*	2.8	0.013
COL4A5	0.3	0.011	*PLA2G7*	0.1	0.041
CSF1	1.5	0.003	*PLCD1*	0.7	0.021
CSF2	2.5	0.042	*PLCE1*	0.4	0.009
CSF3	17.5	0.006	*PLCG2*	472.1	0.01
CXCL10	85.7	0.005	*PTGIR*	6.4	0.043
EDN1	0.3	0.023	*SKI*	0.6	0.018
FAS	1.6	0.019	*SMAD3*	0.6	0.016
FN1	0.3	0.028	*SMAD7*	0.4	0.031
ICAM1	13.9	0.002	*TFRC*	0.6	0.04
ICAM1	22.6	0.002	*TGFB1*	0.6	0.009
IKBKB	0.8	0.037	*TNFRSF18*	1.6	0.007
IL12A	6.3	0.005	*TNFRSF1A*	0.7	0.014
IL1A	11.1	0.02	*TNFRSF1B*	2.1	0.045

* Paired T-test by StatMiner.

Supplemental Material, Table 6. Ingenuity toxicity list of functions and pathways relevant to asbestos exposure, based on the differential expression of 32 genes in SAE cells following 48 h asbestos treatment.

**Ingenuity Toxicity Lists**	**Ratio**	**P Value**	**Molecules Involved**
Hepatic Fibrosis	0.11	0.0000015	*VEGFA, IL8, IL1A, ICAM1, FN1, CCL2, CSF1, IL6, TNF*
LXR/RXR Activation	0.09	0.00021	*IL1A, CCL2, NFKB2, IL6, TNFRSF1B, TNF*
Hepatic Stellate Cell Activation	0.14	0.00027	*IL8, CCL2, NFKB2, IL6, TNF*
Hepatic Cholestasis	0.04	0.0012	*IL8, IL1A, NFKB2, IL6, TNFRSF1B, TNF*
Pro-Apoptosis	0.10	0.0027	*BAX, TNFRSF1B, TNF, FAS*
NFκB Signaling Pathway	0.04	0.0036	*IL1A, LTA, NFKB2, TNFRSF1B, TNF*
Oxidative Stress	0.07	0.0047	*ICAM1, NFKB2, IL6, TNF*
Aryl Hydrocarbon Receptor Signaling	0.03	0.0067	*IL1A, BAX, NFKB2, IL6, TNF*
Mechanism of Gene Regulation by Peroxisome Proliferators via PPARα	0.04	0.011	*IL1A, NFKB2, TNFRSF1B, TNF*
PPARα/RXR Activation	0.02	0.029	*PLCD1, PLCE1, NFKB2, IL6*
PXR/RXR Activation	0.03	0.12	*IL6, TNF*
FXR/RXR Activation	0.02	0.14	*IL1A, TNF*
p53 Signaling	0.02	0.15	*BAX, BCL2*
RAR Activation	0.02	0.21	*VEGFA, NFKB2*
LPS/IL-1 Mediated Inhibition of *RXR* Function	0.01	0.28	*TNFRSF1B, TNF*
Positive Acute Phase Response Proteins	0.03	0.29	*HMOX1*
Anti-Apoptosis	0.03	0.29	*BCL2*
Hypoxia-Inducible Factor Signaling	0.01	0.41	*VEGFA*
TR/RXR Activation	0.01	0.44	*ADRB1*
Oxidative Stress Response Mediated by *Nrf2*	0.005	0.62	*HMOX1*

Note: IPA-Tox analysis allows assessing the toxicity of asbestos by generating a list of tox functions and/or pathways relevant to asbestos exposure. Ratio = the number of "genes of interest" in the current study that belong to a given pathway, divided by the total number of genes that make up the pathway (obtained from the existing literature); for example, a ratio of 0.025 indicates that 2.5% of the total gene molecules in a given pathway were also found in this study. **P-value:** the p-value tells the significance of the association between a specific pathway and the genes of interest in this study.

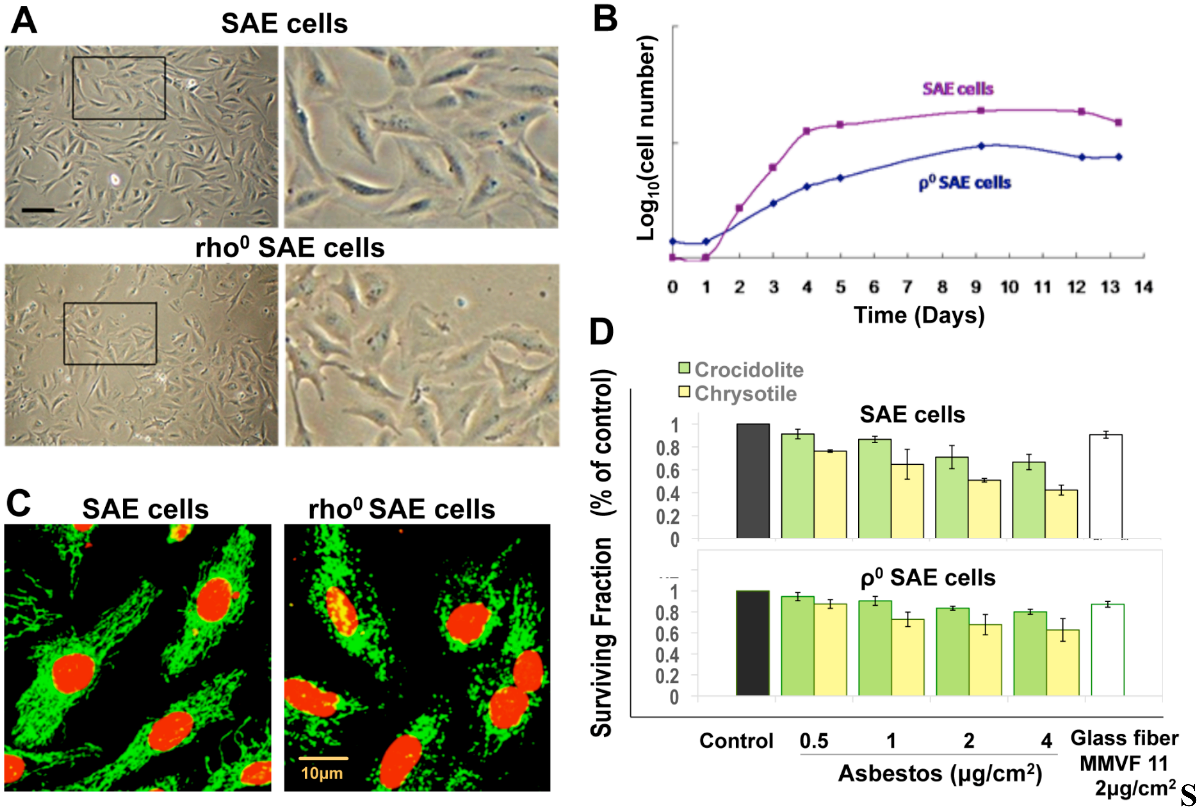

Supplemental Material, Figure 1. Characterization of parental and ρ^0 SAE cells. **A,** the morphology of parental and ρ^0 SAE cells under optic microscope. *Bar,* 50µm. *Right,* enlarged image of boxed area (*left*). **B**, growth curves of parental and ρ^0 SAE cells. The population doubling time of each cell line was consistent for cells at different passages. **C**, mitochondrial morphology. The mitochondria are fragmented in ρ^0 cells but elongated and filamentous in parental cells. The green fluorescence indicates mitochondria stained with an antibody against pyruvate dehydrogenase (PDH), a mitochondrial enzyme. *Bar*, 10 µm. Images were captured with fluorescence microscopy (Olympus Bh-2 equipped with Olympus MicroSuite FIVE software). **D**, Toxicity of chrysotile and crocidolite asbestos fibers on ρ^0 and parental SAE cells. The cellular toxicity of asbestos was indicated by survival fraction (percentage of survival over untreated controls) measure with the CyQuant cell proliferation Assay kit. Asbestos and glass fibers exposure duration: 72 hours. Data (mean ± SD) from average of three independent experiments.

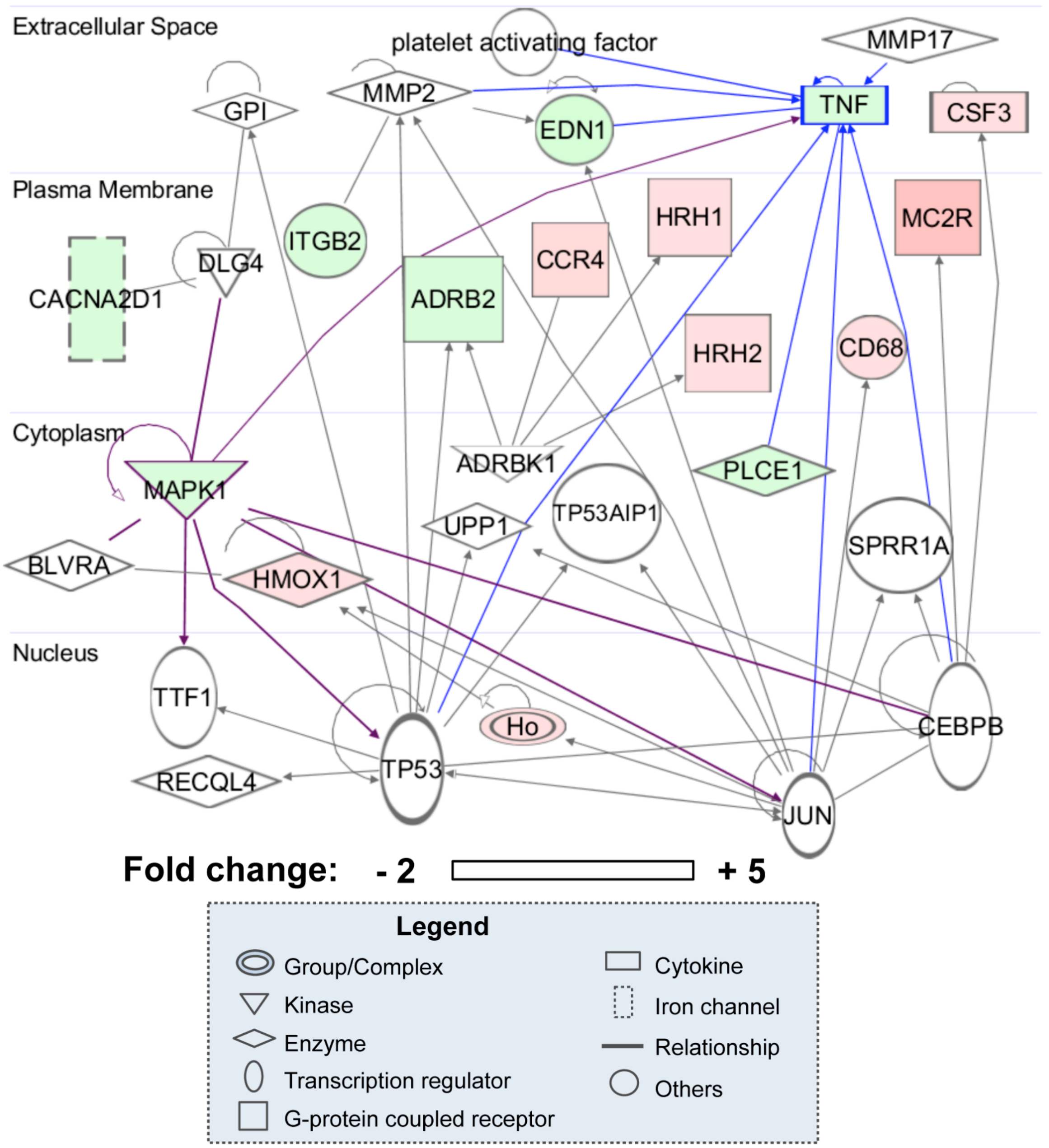

Supplemental Material, Figure 2. Network of genes mediated by asbestos after 48 hours of treatment in ρ^0 SAE cells. A highly interconnected network of 12 asbestos-responsive genes ($P<0.05$) was constructed by IPA, based on direct interactions stored in the Ingenuity Knowledge Base, which is a collection of experimentally confirmed relationships between molecules. Color scale bar indicates the fold change in gene expression level, green: down regulation, red: up regulation. Un-colored molecules: expression levels were not examined in the current study. **Note:** "Ho" is a group/complex suggested by IPA to contain gene(s) examined by our study. "*TNF*" and "*HRH2*" were not significantly down- and up- regulated by asbestos according to StatMiner (P value = 0.03 and 0.26, respectively; but suggested by IPA to be affected by asbestos treatment based on the input of 12 interconnected asbestos-responsive genes (this Figure and Supplemental Material, Table 4).

References

King MP, Koga Y, Davidson M, Schon EA. 1992. Defects in mitochondrial protein synthesis and respiratory chain activity segregate with the tRNA(Leu(UUR)) mutation associated with mitochondrial myopathy, encephalopathy, lactic acidosis, and strokelike episodes. Mol Cell Biol 12(2): 480-490.

King TE, Ronald WE, Maynard EP. 1967. [58] Preparation of succinate dehydrogenase and reconstitution of succinate oxidase. In: Methods in Enzymology: Academic Press, 322-331.

Liu SX, Davidson MM, Tang X, Walker WF, Athar M, Ivanov V, et al. 2005. Mitochondrial damage mediates genotoxicity of arsenic in mammalian cells. Cancer Res 65(8): 3236-3242.

Partridge MA, Huang SXL, Hernandez-Rosa E, Davidson MM, Hei TK. 2007. Arsenic Induced Mitochondrial DNA Damage and Altered Mitochondrial Oxidative Function: Implications for Genotoxic Mechanisms in Mammalian Cells. Cancer Res 67(11): 5239-5247.

Reers M, Smiley ST, Mottola-Hartshorn C, Chen A, Lin M, Chen LB. 1995. Mitochondrial membrane potential monitored by JC-1 dye. Methods Enzymol 260: 406-417.

Salviati L, Hernandez-Rosa E, Walker WF, Sacconi S, DiMauro S, Schon EA, et al. 2002. Copper supplementation restores cytochrome c oxidase activity in cultured cells from patients with SCO2 mutations. Biochem J 363(Pt 2): 321-327.

Shao G, Berenguer J, Borczuk AC, Powell CA, Hei TK, Zhao Y. 2006. Epigenetic inactivation of Betaig-h3 gene in human cancer cells. Cancer Res 66(9): 4566-4573.

Zhou H, Ivanov VN, Lien Y-C, Davidson M, Hei TK. 2008. Mitochondrial Function and Nuclear Factor-{kappa}B-Mediated Signaling in Radiation-Induced Bystander Effects. Cancer Res 68(7): 2233-2240.
